# COVID-19 rapid response in a limited resource setting (notes from the field): Chinhoyi Provincial Hospital, Mashonaland West Province, Zimbabwe

**DOI:** 10.11604/pamj.2021.39.111.29241

**Published:** 2021-06-08

**Authors:** Munashe Chimene, Leroy Gore Nhari, Mathias Dzobo, Malizgani Mhango, Tafadzwa Dzinamarira

**Affiliations:** 1 COVID-19 Response Team, Chinhoyi Provincial Hospital, Chinhoyi, Zimbabwe,; 2 National Tuberculosis Reference Laboratory, Bulawayo, Zimbabwe,; 3 College of Health Sciences, Faculty of Medicine, University of Zimbabwe, Harare, Zimbabwe,; 4 School of Public Health, University of Western Cape, 7535, Cape Town, South Africa,; 5 Department of Public Health Medicine, School of Nursing and Public Health, University of KwaZulu-Natal, Durban, 4001, South Africa,; 6 School of Health Systems and Public Health, University of Pretoria, Pretoria, 0002, South Africa

**Keywords:** COVID-19, response, surveillance, limited resources

## Abstract

COVID-19 has impacted health systems globally with varying impacts across regions. In Zimbabwe, a country with perennial problems of shortage of healthcare workers and resources, the pandemic has caused substantial strain on the public health system. The ability to share experiences on what has worked and what has not can be valuable as scientists, policymakers, and others determine steps forward and reflect backward to determine lessons learned in the pandemic response. We describe the setup and function of a COVID-19 rapid response team in the context of a limited resource setting. The response had to be tailored to make maximal use of the resources available and manage the outbreak. In this article, we share notes from the field and discuss the process of setting up a rapid response protocol in a limited resource provincial hospital, the challenges encountered, improvised interventions and recommendations for managing a COVID-19 resurgence and future similar pandemics.

## Perspective

Since the last quarter of 2019, when the first case of COVID-19 was reported, there have been over 116 million cases and over 2.5 million deaths globally as of 6^th^ March 2021 [1]. On the same date, over 3,9 million cases and over 105,000 deaths have been reported in Africa with Zimbabwe reporting 36,260 cases and 1,485 deaths [2]. The impact of the pandemic in Africa has been substantially lower than what earlier models predicted [3]. However, the effects on the health system and people´s livelihoods have been far-reaching. In Zimbabwe, the pandemic has caused unprecedented socio-economic challenges and disruption of provision of healthcare services implications on the population [4]. As the COVID-19 outbreak broke, functional, trained and well-equipped rapid response teams (RRTs) of diverse technical skills and competencies were the cornerstone of any response strategy [5]. Major contributions of the RRT were proffering solutions and devising strategies that aided in ameliorating the effects of the COVID-19 pandemic including reduction of mortality rates. RRTs would perform rapid assessment of the suspected COVID-19 outbreak through case identification and confirmation and establishing the descriptive epidemiologic picture of the outbreak. Additionally, RRTs functioned as counselors to those COVID-19 patients and contacts of confirmed cases in isolation and quarantine respectively. In Mashonaland West Province, a RRT was formed at Chinhoyi Provincial Hospital.

Mashonaland West province is home to over 1,5 million people (11.5% of the Zimbabwean population) [6]. As of 6^th^March 2021, the province has reported 2,037 COVID-19 cases with 133 deaths [7]. The province has seen two COVID-19 waves; August 2020 and January 2021. Geographically, the province has an area of 57,441 km^2^. Mashonaland West Province comprises seven districts namely Chegutu, Hurungwe, Kariba, Makonde, Mhondoro-Ngezi, Sanyati and Zvimba. There are seven district hospitals, four mission hospitals and five rural hospitals in the province. The referral hospital in the province, Chinhoyi Provincial Hospital which is a tertiary level referral centre is located in Chinhoyi town which is in Makonde district. This paper will focus on the set up of a rapid response protocol at Chinhoyi Provincial Hospital, discuss challenges encountered and improvised interventions. The paper will also discuss recommendations for managing a COVID-19 resurgence and future similar pandemics in limited resource settings. COVID-19 was the first major pandemic since the 2008 cholera outbreak. The set-up of the provincial COVID-19 rapid response team (RRT) in Mashonaland West Province was premised on the experience from the 2008 cholera outbreak in the province. The response team had to leverage expertise gained in the control of the 2008 cholera outbreak.

### Challenges and improvised interventions

Setting up a rapid response team in an institution that had no formal emergency response protocols and guidelines came with its challenges. With limited resources at the hospital´s disposal, the RRT had to come up with a functional outline on how to proceed and set out reasonable objectives. Table 1 shows some of the challenges encountered and improvisations made to surmount them.

**Table 1:** presentatio of challenges and improvised interventions

	Challenges encountered	Improvised interventions
Human resources	- Limited staff. The number of posts for the various health workers at Chinhoyi Provincial Hospital had not been revised for the past 20 years despite the hospital expansion. This posed a great challenge in terms of work burden. This also resulted in low staff morale	- Increased working hours - Creation of a parallel working shift schedule. Nurses were assigned to more than one task i.e ward duties and testing duties - Exclusion of 2 doctors who were incorporated in the RRT from main hospital duties i.e. outpatient departments, on-call duties, minor surgical procedures to focus on COVID-19 tasks
Administrative	- Limited budget for COVID-19 response	- The hospital came up with a budget outside of the usual government allocation - Introduced testing times for COVID-19 to properly manage available personal protective equipment (PPE)
Infrastructure and equipment	- There was no dedicated infectious diseases ward ; - There was no PCR machine - There was no existing structure to set up sample collection booths	- A dysfunctional renal unit was turned into a makeshift COVID-19 isolation ward. Secondly, one of the functional main wards was converted into a COVID-19 isolation ward - The Cepheid Gene Xpert machine, used for TB testing was split between COVID-19 and TB - Due to limited resources, tents were set up for patient screening and sample collection
Case management	- Lack of comprehensible national guideline of management of COVID-19 at the time of setting up the rapid response team	- Rapid development of an in-house guideline adopted by the hospital administration
Communication	- Lack of an established communication process on health worker deployment	- Utilization of social media such as WhatsApp groups where health workers of different cadres would interact and coordinate duties - Establishment of weekly meetings to review the previous week´s activities

### Recommendations for managing a COVID-19 resurgence and future similar pandemics

#### Recommendations for Chinhoyi Provincial Hospital

COVID-19 will not be the last pandemic that Zimbabwe will have to deal with. Lessons can be learnt from the experiences in handling the current pandemic to inform future responses. Some of the countries which have had considerable success in containing the pandemic, with relatively less mortality and negative economic consequences, like South Korea and China, have learnt and addressed the weaknesses revealed by past pandemics like severe acute respiratory syndrome (SARS) in 2003 and Middle East respiratory syndrome (MERS) in 2015. There is a need to do away with a panic-forget approach and invest in health systems strengthening in Zimbabwe to improve preparedness in handling future pandemics.

### Health workforce considerations

This pandemic has changed how health workers provide care, are deployed and managed. It has exposed the consequences of the lack of attention to health workforce considerations and how necessary these are for improved pandemic preparedness and responsiveness. There is a need for the government and relevant stakeholders to incorporate options for scaling up the workforce in outbreaks and pandemics by utilizing modeling and scenario planning. The current national response to COVID-19 can serve as a template upon which response for future pandemics can be modeled on. Sufficient financial resources are needed to support such workforce projections and other requirements through training in outbreak response. Furthermore, the welfare of healthcare workers will need to be prioritised, unlike the experiences that characterised the response to the current COVID-19 pandemic. The hospitals should have robust procurement structures to bring PPE that protects healthcare workers from occupational exposure.

### Health information systems

Health information systems (HIS) developed to handle the current pandemic should be strengthened and improved to enhance preparedness for future pandemics. Previously, Zimbabwe had not had to deal with a countrywide outbreak that required effective information dissemination and data sharing to all parts of the country. The current pandemic provides an opportunity to leverage the communication infrastructure that was used to collect, analyse, and distribute data to inform day to day decision making. A strong and effective HIS that is adequately funded and supported by the central government before a future outbreak can strengthen health systems and pandemic preparedness and response capacities in the case of a future countrywide pandemic.

### Infrastructure

Further, we recommend the government to continue developing diagnostic infrastructure especially in provinces outside Harare. The initial response to the pandemic was marked by low rates of screening and testing for COVID-19 due to shortages and centralization of diagnostic infrastructure and expertise in the capital city. Secondly, the public health laboratories should be capacitated with modern diagnostic machines and personnel have to be trained to perform advanced diagnostic molecular techniques such real-time polymerase chain reaction (PCR) [8]. A prominent feature of the response to this pandemic was the lack of ventilators in major government hospitals. We recommend that the government allocate resources to acquire ventilators which not only serve COVID-19 patients but any other health conditions that may need breathing support to patients. Specifically, for Chinhoyi Provincial Hospital, the government must support establishment of a permanent infectious disease ward in the hospital that is fully equipped with latest equipment and staff in case of a pandemic. This ward may be a good start to serve as the infectious disease treatment center for the province.

### Formulation of a disaster management plan and establishment of standard operating procedures

The current pandemic has revealed the inadequacy or lack of sound health emergency response guidelines and the will to implement them. Our recommendation to the Zimbabwean government is to craft guidelines for responding to a health emergency or outbreak which is multisectoral as evidenced by how COVID-19 pandemic almost halted every aspect of life when lockdowns were introduced. There is a need to formulate health specific guidelines that address human resources allocation and protection of frontline workers, diagnostic provisions, supply chain considerations, communication and awareness as well as data collection and analysis. These guidelines will always have to be adjusted to suit the pandemic or outbreak that will be prevailing but on the overall it promotes a rapid response compared to not having guideline at all.

### Improved management of health workers to address a crisis

RRT members need to be viewed as deployable assets which can always be deployed to the area of need in a public health emergency. There is need for an established code of conduct for RRT members´ needs to be formulated. One major hurdle in the work of RRT at Chinhoyi Provincial Hospital was the reluctance to move cadres from their usual duties to an area of public emergency. All health care worker cadres have to be conscientized to the fact that they are deployable in cases of public health emergencies.

### Administrative considerations

It is well established elsewhere that salaries for health care workers in Zimbabwe are very low [9]. There is an urgent need for the government to address health care worker remuneration related grievances to improve morale and support the immediate pandemic response. This goes a long way in motivating health care workers. Secondly, the current bureaucracy in the distribution of PPE remains a challenge in the delivery of service at Chinhoyi Provincial Hospital. There is need for streamlining procurement and distribution systems to better coordinate supplies management.

### Recommendations for other low-income settings

The initial COVID-19 response in many low-income countries was characterized by a myriad of challenges that emanated from the socio-economic status and geopolitical environment of these countries. This humanitarian tragedy brought stress and strain both across individuals and institutions, and the response brought with it seismic shifts in healthcare delivery both in where healthcare services are provided and how they are delivered. However, the experience of the COVID-19 pandemic may provide room for significant improvements in the establishment of RRTs, design of health facilities, training of healthcare workers, sourcing and inventory management of medical supplies and critical care equipment. In this sub-section, we discuss considerations for health systems in low-income countries to strengthen out break response systems.

### Continual investment in and support of rapid response teams

There is need for investment in and support of rapid response teams in low-income countries. The coordination of RRTs for the COVID-19 response in low-income countries revealed a lack of continual support as evident by the lack of rosters with current contact details of the team members resulting in delayed deployment of the RRTs. The importance of planning to preemptively address such challenges has been well covered elsewhere [10]. Continual investment in RRTs would allow for continual update of the details of all team members and regular training. Regular training prepares the team members for future emergencies by keeping them abreast with new technologies and skills.

### Accelerating technology in healthcare delivery systems

In preparation for future pandemics, it is important that established traditional methods of delivering facility-based healthcare be revisited and revolutionized to suit the needs of patients in situations that restrict movement such as lockdowns. Technologies such as telemedicine should be supported. Despite the plethora of evidence on the benefits of telemedicine, there has been limited investment in this technology in developing countries. We recommend investment in telemedicine to provide healthcare for routine and chronic patients in times were there is limited access to health facilities due to public health emergencies. Long term scale-up of telemedicine could address other current challenges in health systems of low-income countries including health worker shortages.

### Revolutionize infrastructure at health facilities

During the pandemic there has been enactment of make shift clinics and hospitals globally where warehouses, abandoned buildings and hotels were converted into COVID-19 treatment facilities. This prompts the need for focusing future design and construction of health infrastructure on agility. Health buildings should be flexible enough to allow repurposing of beds to suit the requisite needs in times of pandemics. Due to the infectious nature of COVID-19 the confirmed positive patients were isolated to avoid infecting other patients therefore; future hospitals should maximize infection prevention and control strategies through provision of rooms that house single patients. Further, there is need to integrate the intensive/critical care into the hospital layouts and use-experience with the ability to quickly convert regular beds into intensive beds.

In conclusion, functional, trained and well-equipped RRTs of diverse technical skills and competencies are the cornerstone of any pandemic response strategy. Based on our experience at Chinhoyi Provincial Hospital, we recommend various interventions for managing a COVID-19 resurgence and future similar pandemics in limited resource settings. Limited resources countries need to invest in continual support for RRTs, accelerating technology in the health care delivery and revolutionizing infrastructure at health facilities.
